# A recurrent neural network for rapid detection of delivery errors during real-time portal dosimetry

**DOI:** 10.1016/j.phro.2022.03.004

**Published:** 2022-04-20

**Authors:** James L. Bedford, Ian M. Hanson

**Affiliations:** Joint Department of Physics, The Institute of Cancer Research and The Royal Marsden NHS Foundation Trust, London SM2 5PT, UK

**Keywords:** In vivo dosimetry, Electronic portal imaging device, Artificial neural network, Volumetric modulated arc therapy

## Abstract

**Background and purpose:**

Real-time portal dosimetry compares measured images with predicted images to detect delivery errors as the radiotherapy treatment proceeds. This work aimed to investigate the performance of a recurrent neural network for processing image metrics so as to detect delivery errors as early as possible in the treatment.

**Materials and methods:**

Volumetric modulated arc therapy (VMAT) plans of six prostate patients were used to generate sequences of predicted portal images. Errors were introduced into the treatment plans and the modified plans were delivered to a water-equivalent phantom. Four different metrics were used to detect errors. These metrics were applied to a threshold-based method to detect the errors as soon as possible during the delivery, and also to a recurrent neural network consisting of four layers. A leave-two-out approach was used to set thresholds and train the neural network then test the resulting systems.

**Results:**

When using a combination of metrics in conjunction with optimal thresholds, the median segment index at which the errors were detected was 107 out of 180. When using the neural network, the median segment index for error detection was 66 out of 180, with no false positives. The neural network reduced the rate of false negative results from 0.36 to 0.24.

**Conclusions:**

The recurrent neural network allowed the detection of errors around 30% earlier than when using conventional threshold techniques. By appropriate training of the network, false positive alerts could be prevented, thereby avoiding unnecessary disruption to the patient workflow.

## Highlights

• Real-time portal dosimetry can detect errors in volumetric modulated arc therapy.
• Neural networks avoid false positive errors during intrafraction portal dosimetry.
• Error detection is 30% earlier with an artificial neural network than with thresholds.

## 1 Introduction

Portal dosimetry is widely used to ensure the dosimetric accuracy of radiotherapy delivery [1], [2], [3], [4]. In the case of forward-projection, portal images are predicted at the time of treatment planning, and then measured images are compared with these [5], [6], [7], and in the case of back-projection, measured images are projected onto the CT scan of the patient and converted into a dose distribution, which is then compared with the planned dose distribution [8], [9], [10], [11], [12]. Groups of images are selected to represent the segments of volumetric modulated arc therapy (VMAT) [13], [14].

Usually, images for completed fractions of treatment are analysed. However, there is growing interest in analysing the measured images as the treatment fraction proceeds. In this way, it is possible to identify errors before significant dosimetric impact occurs for the patient [15], [16], [17], [18], [19], particularly for hypofractionated treatments [20], which are becoming increasingly commonplace [21], [22], [23]. The real-time method is time-resolved, which also has its own advantages in giving a more thorough analysis than when using integrated images or dose [24], [25]. Typically, errors are detected by setting a series of thresholds for a number of image features or measures, and then watching for the measures to exceed the thresholds [26], preferably avoiding false positives, which are disruptive in the real-time context [27].

Use of an accurate prediction model is an important means of providing sensitivity to errors while avoiding false positives. However, another possible means of increasing reliability is to use an artificial neural network. Simple neural networks have been used in the radiotherapy context before, such as for prediction of biological outcomes [28] and for pre-treatment quality assurance [29], and more complex neural networks are increasingly used in radiotherapy for deep learning in structure delineation and treatment planning [30], [31], [32], [33]. However, they have so far not been used in the context of error detection in portal dosimetry.

This study therefore investigated the training of a simple artificial neural network to detect errors based on the supplied image measures at each time point. The study was a proof of principle of a recurrent neural network (RNN) approach, using VMAT treatment of the prostate as an illustration.

## 2 Materials and methods

There were several types of neural network that could be used for this application, but the RNN was used in this study because it could not only learn from training data, but also had the ability to learn from, and adapt to, a temporal series of inputs, such as the image measures at each segment of a VMAT arc.  The study used the forward-projection method of portal dosimetry and a variety of deliberate errors. The differences between the measured and predicted images were investigated firstly using multiple separate metrics (MSM) and related thresholds and then with the use of an RNN, so as to quantify the timeliness with which each method was able to detect the errors.

### 2.1 Patients and treatment plans

**Fig. 1:**
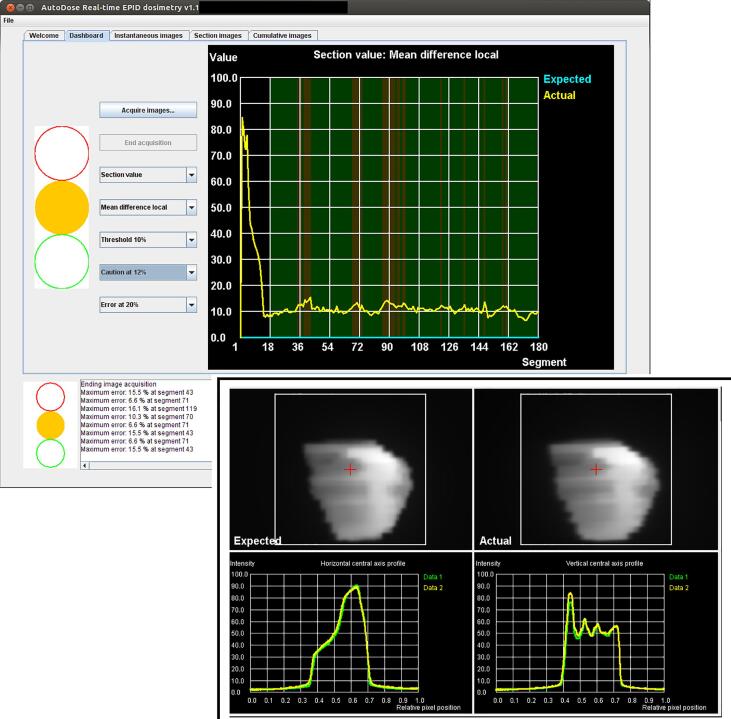
An analysis of a volumetric modulated arc therapy treatment plan for a patient delivery, seen in AutoDose v1.1. The main panel shows the mean image difference as a percentage of local image intensity for sections of arc consisting of 10 segments. The inset (lower right) shows the expected and actual images for a single section of arc, together with horizontal and vertical profiles through the central axis (Data 1 – expected image, Data 2 – actual image).

.eatment plans for radiotherapy of the prostate were created using AutoBeam v5.8 [34] for 60 Gy in 20 fractions with the 6 MV beam of a VersaHD linear accelerator (Elekta AB, Stockholm, Sweden) [35], [36]. For six patients who gave their consent for their images to be used for research, predicted portal images were retrospectively produced for each segment of the VMAT arcs and input to AutoDose v1.1 software for comparison with real-time images [19] (Fig. 1). AutoBeam was also used to recalculate the plans and predicted images on a water-equivalent phantom of dimensions 300 mm long (G-T direction) × 300 mm wide (A-B direction) × 200 mm high, with the isocentre located at the centre of the phantom.

### 2.2 Measured images

Errors were deliberately introduced into all 180 segments of the treatment plans and both the normal and erroneous plans were then delivered to a Solid Water phantom (Radiation Measurements, Inc., Middleton, WI). The errors consisted of a 2–10% increase in monitor units in 2% steps, a retraction of 2–10 mm in 2 mm steps of all multileaf collimator (MLC) leaves, a shift of 2–10 mm in 2 mm steps of all MLC leaves, and introduction of an air space of 10–50 mm width in 10 mm steps into the phantom to simulate rectal gas [37]. In three patients, all error cases were simulated, and in a further three patients, only the error-free case and 4% increase in monitor units, 4 mm MLC retraction, 4 mm MLC shift and 20 mm air space were simulated. Portal images were recorded using an iViewGT imaging panel (Elekta) and analysed using AutoDose, which allocated the images to control points of the treatment plan [19].

### 2.3 Image metrics and selection of thresholds

At each segment of the VMAT plan, four measures of agreement between predicted and measured images were calculated: central axis signal, mean image value, root-mean-square difference as a percentage of global maximum and root-mean-square difference as a percentage of local prediction. These simple difference measures were used in favour of more complex difference measures as the intention was to identify differences, however small spatially or temporally, and then to use error detection to work with these. The first 10% of segments were neglected as the images were not stable in this period. The startup of the linear accelerator, estimated to affect the first 1% of segments, may have been contributory to this instability. After the first 10% of segments, a running sum of 10 segments was used. For comparison purposes MSM was applied, in which the value of median + 2 × range of the maximum value of each statistic over the cases under consideration was taken as the threshold, and image metrics exceeding these thresholds signified errors.

### 2.4 Recurrent neural network

The four measures were applied to an RNN [38] consisting of four layers of gated recurrent units (GRUs), with four nodes in the first layer, eight in the second layer, four in the third layer and one in the final layer. The function of the GRU was exactly as defined by Cho et al. [39]. For training and testing, a leave-two-out cross-correlation strategy was used [40], [41]. Four of the patients were used to train the network, and the remaining two patients were used to test the result. Of the four patients used for training, two were from patients 1–3, for which a full set of error cases were available, and the other two were from patients 4–6, for which only representative errors were available (see section 2.2). There were therefore nine ways of selecting unique combinations of patient for testing, so the RNN was trained and tested nine times. For example, firstly patients 1 and 4 were retained for testing, so patients 2, 3, 5, and 6 were used for training. Then patients 1 and 5 were retained for testing, so patients 2, 3, 4 and 6 were used for training, etc.

Using *p* to index the *P* training patients, *e* to index the *E* + 1 error types, (*e* = 0 representing no error), *s* to index segments after exclusion of the first 19 segments and the vector **w** to represent the *W* weights of the RNN, the objective function for training was defined as:

(1) $$f\left(p,e,s,w\right)=\sum_{p=1}^{P}\sum_{e=0}^{E}\sum_{s=1}^{162}f_{0}\left(e\right)\cdot f_{e}\left(e\right)\cdot f_{s}\left(s\right)\cdot f_{y}\left(p,e,s,w\right)+\frac{\lambda }{2W}\sum_{i=1}^{W}w_{i}^{2}$$

The factor $f_{0}\left(e\right)$ was an importance factor to avoid false positives:

(2) $$\begin{array}{cc} f_{0}\left(e\right)= & 10^{-2},\;\;e=0\\ = & 10^{-6},\;\;e=1\ldots E \end{array}$$

and $f_{e}\left(e\right)$ was an error-specific factor to ensure that the larger errors were detected:

(3) $$\begin{array}{cc} f_{e}\left(e\right)= & 1,\;\;\;\;e=0\\ = & 10^{M_{e}-1},\;\;e=1\ldots E, \end{array}$$

where *M_e_* was the physical ranking of the error, i.e. 1 to 5 according to a monitor unit increase of 2% to 10% etc. The factor $f_{s}\left(s\right)$ was a segment-specific factor:

(4) $$\begin{array}{cc} f_{s}\left(s\right)= & \left(163-s\right)/162,\;\;\;e=0\\ = & s/162,\;\;\;\;\;\;\;e=1\ldots E, \end{array}$$

thereby emphasising the importance of early segments in normal cases and late segments in error cases. Finally, $f_{y}\left(p,e,s,w\right)$ provided a quadratic penalty from the “off” state for normal cases and from the “on” state for error cases:

(5) $$\begin{array}{cc} f_{y}\left(p,e,s,w\right)= & {\left[1+y\left(p,e,s,w\right)\right]}^{2},\;e=0\\ = & {\left[1-y\left(p,e,s,w\right)\right]}^{2},\;e=1\ldots E, \end{array}$$

where $y\left(p,e,s,w\right)$ was the output of the network$\left(-1<y<1\right)$, with *y* > 0 signifying an error and *y* < 0 signifying normal delivery.

The final term in equation (1) was an *L*_2_ norm to prevent overfitting to the training data. This was applied to the *W* primary weights of the network, excluding the hidden state, update and reset weights, using an empirically-determined value of 40 for the regularisation parameter, *λ*. To further avoid false positives, indices of *e* for which *M_e_* = 1, i.e. 2% increase in monitor units, 2 mm aperture opening etc, were also defined as normal (no-error) cases. Due to the non-convexity of the objective function, a random search algorithm was used for training. The software was run on a SPARC T4-2 server with 128 hyper-threads (Oracle Corporation) using a separate execution thread for each of the nine combinations of training and testing.

To visualise real-time performance, the network trained on patients 2, 3, 5, and 6 was applied to errors for patient 1. The final validation was to apply the RNN to actual patient images for four patients (A-D) different to those used for the phantom study. All of these treatments were considered to be normal deliveries, but the images for patient D were re-acquired on further occasions (in a non-real-time workflow) and were taken as an example of images that the medical physicist was not satisfied with.

## 3 Results

### 3.1 Training the recurrent neural network

**Fig. 2:**
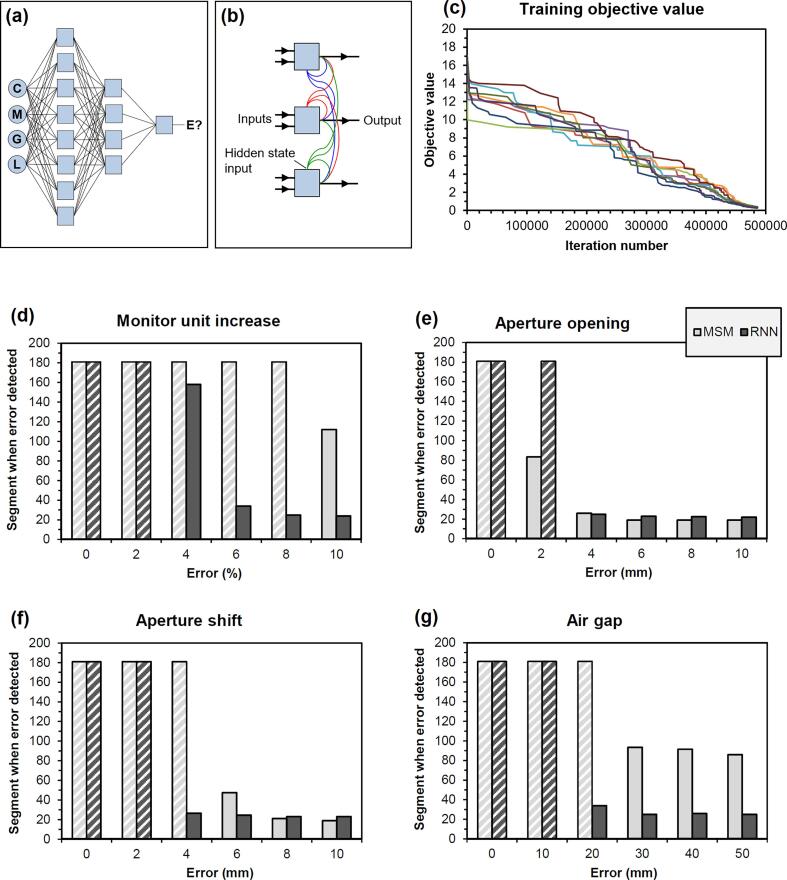
Training the recurrent neural network. (a) Network topology, (b) abstraction of one layer of the network, (c) training progress for the nine data sets, (d)-(g) Median index of the first segment at which each error is detected, as a function of error type and magnitude. White cross-hatching indicates that the error is not detected. C: central image signal, M: mean image value, G: root-mean-square error as a percentage of global maximum, L: root-mean-square error as a percentage of local signal, E: error, MSM: multiple separate metrics, RNN: recurrent neural network.

Training and testing of the network required around 50 h. Over this time, the training progressed steadily, with the objective function converging to a similar value for the nine data sets (Fig. 2). Benefits were observed in timeliness of error detection with the RNN for monitor unit, aperture shift and air gap errors. Importantly, there were no false positives in any of the error-free cases. For the training cases as a whole, the median segment index at which errors were detected was 105 (range 97 – 120) for MSM and 68 (range 52 – 75) for the RNN, with a median relative reduction of 0.57 (range 0.49 – 0.72). The delivery time was approximately 180 s for the 180 segments of these treatment plans, so in terms of time, each segment equated to approximately 1 s of delivery time. Thus, finding the error at segment 68 meant that approximately 68 s of delivery was completed when the error was detected. There were 186 false negatives, in which the error was not detected at all during the 180 segments, out of 432 errors for MSM, representing a ratio of 0.43. There were 100 false negatives out of 432 errors for the RNN, a ratio of 0.23.

### 3.2 Testing the recurrent neural network

**Fig. 3:**
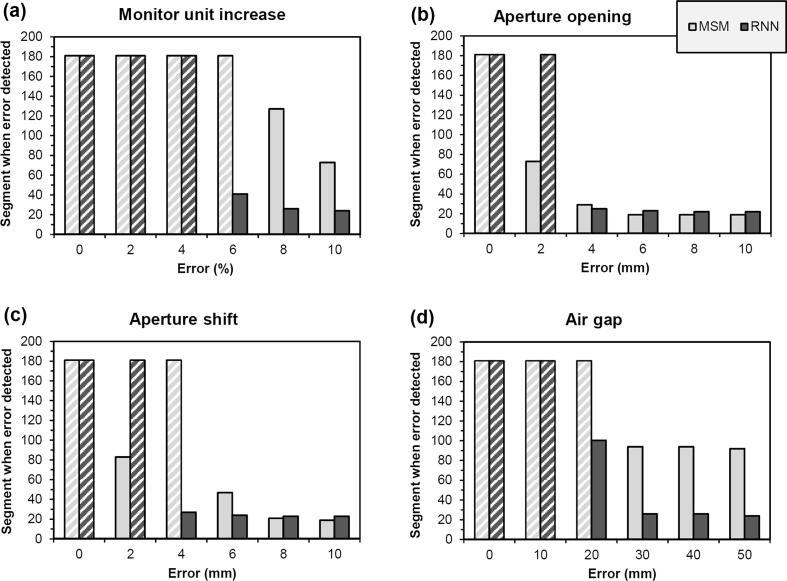
Median index of the first segment at which each error is detected, as a function of error type and magnitude, during testing. White cross-hatching indicates that the error is not detected. MSM: multiple separate metrics; RNN: recurrent neural network.

Testing showed that the RNN was most beneficial for errors in monitor units, aperture position and path length (Fig. 3). MSM were already effective in detecting errors in aperture opening, so in this case the RNN was less beneficial. The thresholds for central image signal and mean image value were exceeded in several instances for an aperture shift of 2 mm (Fig. 3c) but not for 4 mm, unrelated to the errors being introduced. The slightly worse performance of the RNN for larger aperture opening and aperture shift errors (Fig. 3b and 3c) was due to the *L*_2_ norm. This prevented overfitting, but meant that some of the obvious errors were not found until several segments after the MSM method.

**Fig. 4:**
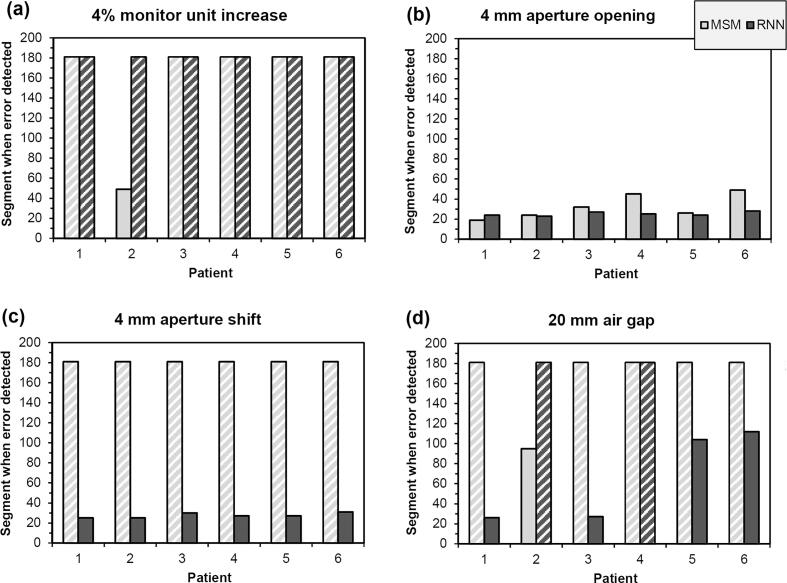
Index of the first segment at which each error is detected, in the six patients separately, for a fixed level of error, during testing. White cross-hatching indicates that the error is not detected. MSM: multiple separate metrics; RNN: recurrent neural network.

Testing results for a specific level of error were found to be broadly similar between patients (Fig. 4), although overall, there was some variation in the nine test samples (Table 1). Again, there were no false positives in any of the test results for error-free cases. There were 77 false negatives out of 216 errors for MSM, representing a ratio of 0.36. There were 52 false negatives out of 216 errors for the RNN, a ratio of 0.24.

**Table 1:** Mean segment index at which errors are detected for multiple separate metrics with threshold and for a recurrent neural network, during testing.

Patient A	Patient B	Error Size*	MSM	RNN	Relative benefit†
1	4	Small	159	181	1.14
		Medium	129	38	0.29
		Large	78	23	0.29
		**Overall**	**117**	**57**	**0.49**
1	5	Small	159	105	0.66
		Medium	120	51	0.43
		Large	78	23	0.29
		**Overall**	**113**	**51**	**0.45**
1	6	Small	159	142	0.89
		Medium	130	60	0.46
		Large	78	23	0.29
		**Overall**	**117**	**62**	**0.53**
2	4	Small	114	181	1.59
		Medium	84	84	1.00
		Large	40	33	0.83
		**Overall**	**74**	**83**	**1.12**
2	5	Small	114	151	1.32
		Medium	92	61	0.66
		Large	38	32	0.84
		**Overall**	**78**	**66**	**0.85**
2	6	Small	115	103	0.90
		Medium	78	77	0.99
		Large	42	24	0.57
		**Overall**	**72**	**63**	**0.88**
3	4	Small	129	181	1.40
		Medium	131	72	0.55
		Large	59	74	1.25
		**Overall**	**107**	**74**	**0.69**
3	5	Small	129	181	1.40
		Medium	122	66	0.54
		Large	58	24	0.41
		**Overall**	**102**	**71**	**0.70**
3	6	Small	129	181	1.40
		Medium	131	80	0.61
		Large	59	24	0.41
		**Overall**	**107**	**78**	**0.73**
**MEDIAN**		**Overall**	**107**	**66**	**0.70**

MSM: multiple separate metrics; RNN: recurrent neural network.

* Small: 2% monitor unit increase, 2 mm aperture opening, 2 mm aperture shift, 10 mm air gap; medium: 4–6% monitor unit increase, 4–6 mm aperture opening, 4–6 mm aperture shift, 20–30 mm air gap; large: 8–10% monitor unit increase, 8–10 mm aperture opening, 8–10 mm aperture shift, 40–50 mm air gap.

† Relative benefit defined as quotient of RNN and MSM.

**Fig. 5:**
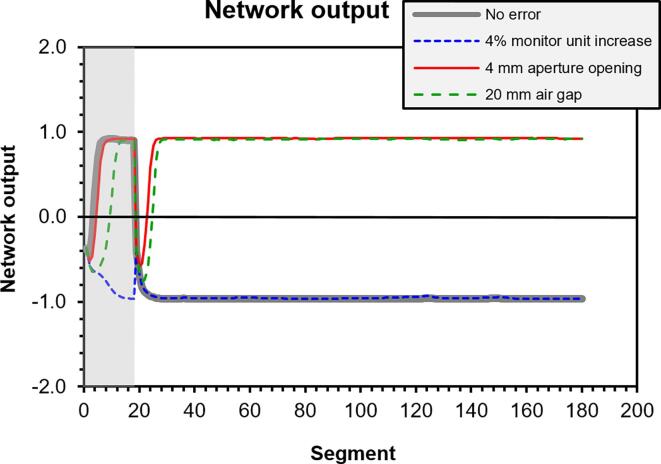
Network output for patient 1 for several error cases. Results less than or equal to zero indicate absence of an error and results greater than zero indicate an error. The output in the grey region at the left is disregarded due to instability of the raw signals.

In the real-time context, the RNN was found to be most active initially in the treatment delivery for the case of moderate errors (Fig. 5). The network failed to detect a 4% increase in monitor units (Fig. 4a), but successfully detected the other errors rapidly (Fig. 4b-d). After error detection, the signal did not change appreciably.

For the real patient images, deliveries for patients A-C were classified as normal, with a network output of close to −1. Those for patient D were identified very rapidly as abnormal, with the network output quickly moving to approach +1.

## 4 Discussion

The results show that in the context of forward-projection real-time portal dosimetry for prostate treatment delivery, the RNN is able to improve the timeliness of error detection by around 30%, compared to MSM. There is some variability in effectiveness of the RNN between error types and between patients.

Implicitly, the thresholds of MSM are built in to the RNN in the form of the biases, but the more complex connectivity of the RNN is shown to provide a more effective result, similar to dose-volume histogram prediction [42]. The RNN is trained to detect particular types of errors for a particular treatment site, and there is no guarantee that it operates correctly for other errors or treatment sites. In other words, although the *L*_2_ norm prevents overfitting within the patients used, the model as a whole may be over-fitted to certain types of error and treatment site. However, by using general image difference measures, the present study gives an indication of what is likely to be achieved in a larger study using treatment plans of similar complexity.

There are relatively few studies focusing on real-time EPID dosimetry for VMAT, but it is possible to make some comparisons with other studies. The method behaves similarly to that of Woodruff et al. [17], except for the use of section images rather than integrated images. Compared to real-time MSM using site-specific control limits [15], which is able to detect monitor unit errors of 5% in static gantry intensity-modulated radiotherapy after about 23% of the delivery, the detection speed in the present study is slower, but the thresholds must be higher with VMAT due to the gantry rotation, which explains this effect. Monitor unit changes and aperture shifts of a similar magnitude to those in the present study can also be detected by back-projection in a non-real-time context [43], [44]. In the real-time situation, Spreeuw et al. [18] show that a 20 cGy dosimetric difference in the patient can be detected after around 10% of the delivery time for deliberately introduced serious errors in prostate radiotherapy. This is faster than either MSM or RNN in this study, but is expected to be so because of the magnitude of the errors. The study presented here is in agreement with Schyns et al. [25] that the time-resolved element is valuable in the forward-projection approach but that interpretation of any errors detected in terms of dose to the patient is not straightforward.

As with all studies using deliberate errors, the results must either be based on phantom studies or simulated measurements. For the former, used in this study, the anatomy is somewhat simplified, but the measurements include real variations in quality of panel output and calibration. Other uncertainties are the start-up of the accelerator, the initial instability of the images and the allocation of images to segments of the treatment plan. The method of using a running sum of images for a limited number of treatment plan segments is able to detect errors for parts of the VMAT arc, but this has not been fully demonstrated in this study as the introduced errors are present for the whole arc. However, the method of detecting errors in the whole plan does have the advantage that the timeliness of the detection can be quantified in an analogue manner, such as using segment number at which the error is detected, whereas the introduction of short errors means that the detection is binary, for example detected or not, which is then difficult to analyse in small data sets. It is also more important to detect and act upon persistent errors.

Simulated measurements are easier to obtain, by taking predictions and applying noise, e.g. [45], but it is very difficult to ensure that the noise accurately represents the random and systematic errors that typically occur during operation of a portal dosimetry service [46], [47], [48]. In addition, the effectiveness of the portal dosimetry method depends on how accurate the prediction method is [43], [44]. The study does not address patient positioning errors, for which a method such as conebeam CT is more suitable, either separately from the portal dosimetry, or included within it [7], [44], [49]. However, it is likely that anatomical changes can be detected with improved accuracy using the RNN, particularly as this type of change may only impact on the portal images at particular gantry angles [24], [25].

Avoidance of false positive results is an important part of this approach, as a false positive error in the real-time context means that the patient’s treatment is paused while the error is investigated. False positives also add to the operator workload and encourage a lax attitude towards real errors when they occur. There are some false negative results in the study, mostly for the small error cases where the clinical impact is relatively small, but these are reduced in number by appropriate training of the RNN [50].

A logical progression of this work is use a deep learning approach [30], [31], [51], [52] to analyse the predicted and measured images as a whole. Either the pixels of a difference map between the predicted and measured images, or the pixels of both of the images separately could be applied to the inputs. A convolutional stage could detect specific image features which might be indicative of errors.

The RNN presented in this study, taking as input several measures of difference between predicted and measured images, can be used to provide timely indication of errors during real-time portal dosimetry. In this simulation study of forward-projection portal dosimetry for prostate VMAT, a variety of errors are detected around 30% earlier than when using the image difference measures alone in a threshold-based approach. The leave-two-out strategy used in this feasibility study gives an indication of the benefit likely to be observed in a larger cohort of similarly complex VMAT treatments.

## Declaration of Competing Interest

The authors declare that they have no known competing financial interests or personal relationships that could have appeared to influence the work reported in this paper.

## References

[1] van Elmpt W., McDermott L., Nijsten S., Wendling M., Lambin P., Mijnheer B. (2008). A literature review of electronic portal imaging for radiotherapy dosimetry. Radiother Oncol.

[2] Mijnheer B., Beddar S., Izewska J., Reft C. (2013). In vivo dosimetry in external beam radiotherapy. Med Phys.

[3] McCurdy B., Greer P., Bedford J., Mijnheer B. (2018). Clinical 3D dosimetry in modern radiation therapy.

[4] Olaciregui-Ruiz I., Beddar S., Greer P., Jornet N., McCurdy B., Paiva-Fonseca G. (2020). In vivo dosimetry in external beam photon radiotherapy: requirements and future directions for research, development, and clinical practice. Phys Imaging Radiat Oncol.

[5] van Zijtveld M., Dirkx M., Breuers M., de Boer H., Heijmen B. (2009). Portal dose image prediction for *in vivo* treatment verification completely based on EPID measurements. Med Phys.

[6] Chytyk-Praznik K., VanUytven E., vanBeek T.A., Greer P.B., McCurdy B.M.C. (2013). Model-based prediction of portal dose images during patient treatment. Med Phys.

[7] Bedford J.L., Hanson I.M., Hansen V.N. (2014). Portal dosimetry for VMAT using integrated images obtained during treatment. Med Phys.

[8] van Elmpt W.J.C., Nijsten S.M.J.J.G., Dekker A.L.A.J., Mijnheer B.J., Lambin P. (2007). Treatment verification in the presence of inhomogeneities using EPID-based three-dimensional dose reconstruction. Med Phys.

[9] Wendling M., McDermott L.N., Mans A., Sonke J.-J., van Herk M., Mijnheer B.J. (2009). A simple backprojection algorithm for 3D in vivo EPID dosimetry of IMRT treatments. Med Phys.

[10] Mans A., Remeijer P., Olaciregui-Ruiz I., Wendling M., Sonke J.-J., Mijnheer B. (2010). 3D Dosimetric verification of volumetric-modulated arc therapy by portal dosimetry. Radiother Oncol.

[11] McCowan P.M., Van Uytven E., Van Beek T., Asuni G., McCurdy B.M.C. (2015). An in vivo dose verification method for SBRT-VMAT delivery using the EPID. Med Phys.

[12] Van Uytven E., Van Beek T., McCowan P.M., Chytyk-Praznik K., Greer P.B. (2015). Validation of a method for in vivo 3D dose reconstruction for IMRT and VMAT treatments using on-treatment EPID images and a model-based forward-calculation algorithm. Med Phys.

[13] McCowan P.M., McCurdy B.M.C. (2016). Frame average optimization of cine-mode EPID images used for routine clinical in vivo patient dose verification of VMAT deliveries. Med Phys.

[14] Cools R.A.M., Dirkx M.L.P., Heijmen B.J.M. (2017). A novel method for sub-arc VMAT dose delivery verification based on portal dosimetry with an EPID. Med Phys.

[15] Fuangrod T., Greer P.B., Woodruff H.C., Simpson J., Bhatia S., Zwan B. (2016). Investigation of a real-time EPID-based patient dose monitoring safety system using site-specific control limits. Radiat Oncol.

[16] Fidanzio A., Porcelli A., Azario L., Greco F., Cilla S., Grusio M. (2014). Quasi real time in vivo dosimetry for VMAT. Med Phys.

[17] Woodruff H.C., Fuangrod T., Van Uytven E., McCurdy B.M.C., van Beek T., Bhatia S. (2015). First experience with real-time EPID-based delivery verification during IMRT and VMAT sessions. Int J Radiat Oncol Biol Phys.

[18] Spreeuw H., Rozendaal R., Olaciregui-Ruiz I., González P., Mans A., Mijnheer B. (2016). Online 3D EPID-based dose verification: proof of concept. Med Phys.

[19] Bedford J.L., Hanson I.M. (2019). A method to verify sections of arc during intrafraction portal dosimetry for prostate VMAT. Phys Med Biol.

[20] Esposito M., Villaggi E., Bresciani S., Cilla S., Falco M.D., Garibaldi C. (2020). Estimating dose delivery accuracy in stereotactic body radiation therapy: a review of in-vivo measurement methods. Radiother Oncol.

[21] Lukka H.R., Pugh S.L., Bruner D.W., Bahary J.-P., Lawton C.A.F., Efstathiou J.A. (2018). Patient reported outcomes in NRG Oncology RTOG 0938, evaluating two ultrahypofractionated regimens for prostate cancer. Int J Radiat Oncol Biol Phys.

[22] Bezjak A., Paulus R., Gaspar L.E., Timmerman R.D., Straube W.L., Ryan W.F. (2019). Safety and efficacy of a five-fraction stereotactic body radiotherapy schedule for centrally located non-small-cell lung cancer: NRG Oncology / RTOG 0813 trial. J Clin Oncol.

[23] Brunt A.M., Haviland J.S., Wheatley D.A., Sydenham M.A., Alhasso A., Bloomfield D.J. (2020). on behalf of the FAST-Forward Trial Management Group. Hypofractionated breast radiotherapy for 1 week versus 3 weeks (FAST-Forward): 5-year efficacy and late normal tissue effects results from a multicentre, non-inferiority, randomised, phase 3 trial. Lancet.

[24] Persoon L.C., Podesta M., Nijsten S.M., Troost E.G., Verhaegen F. (2016). Time-resolved versus integrated transit planar dosimetry for volumetric modulated arc therapy: patient-specific dose differences during treatment, a proof of principle. Technol Cancer Res Treat.

[25] Schyns L.E.J.R., Persoon L.C.G.G., Podesta M., van Elmpt W.J.C., Verhaegen F. (2016). Time-resolved versus time-integrated portal dosimetry: the role of an object’s position with respect to the isocenter in volumetric modulated arc therapy. Phys Med Biol.

[26] Olaciregui-Ruiz I., Rozendaal R., Mijnheer B., Mans A. (2019). Site-specific alert criteria to detect patient-related errors with 3D EPID transit dosimetry. Med Phys.

[27] Bedford J.L., Hanson I.M. (2021). Optimisation of a composite difference metric for prompt error detection in real-time portal dosimetry of simulated volumetric modulated arc therapy. Br J Radiol.

[28] Gulliford S.L., Webb S., Rowbottom C.G., Corne D.W., Dearnaley D.P. (2004). Use of artificial neural networks to predict biological outcomes for patients receiving radical radiotherapy of the prostate. Radiother Oncol.

[29] Mahdavi S.R., Tavakol A., Sanei M., Molana S.H., Arbabi F., Rostami A. (2019). Use of artificial neural network for pretreatment verification of intensity modulation radiation therapy fields. Br J Radiol.

[30] Sahiner B., Pezeshk A., Hadjiiski L.M., Wang X., Drukker K., Cha K.H. (2019). Deep learning in medical imaging and radiation therapy. Med Phys.

[31] Wang M., Zhang Q., Lam S., Cai J., Yang R. (2020). A review on application of deep learning algorithms in external beam radiotherapy automated treatment planning. Front Oncol.

[32] Nguyen D., Jia X., Sher D., Lin M.-H., Iqbal Z., Liu H. (2019). 3D radiotherapy dose prediction on head and neck cancer patients with a hierarchically densely connected U-net deep learning architecture. Phys Med Biol.

[33] Kearney V., Chan J.W., Haaf S., Descovich M., Solberg T.D. (2018). DoseNet: a volumetric dose prediction algorithm using 3D fully-convolutional neural networks. Phys Med Biol.

[34] Bedford J.L. (2013). Sinogram analysis of aperture optimization by iterative least-squares in volumetric modulated arc therapy. Phys Med Biol.

[35] South C.P., Khoo V.S., Naismith O., Norman A., Dearnaley D.P. (2008). A comparison of treatment planning techniques used in two randomised UK external beam radiotherapy trials for localised prostate cancer. Clin Oncol.

[36] Dearnaley D, Syndikus I, Sumo G, Bidmead M, Bloomfield D, Clark C, et al. Conventional versus hypofractionated high-dose intensity-modulated radiotherapy for prostate cancer: preliminary safety results from the CHHiP randomised controlled trial. Lancet Oncol 2012;13:43-54 (supplementary appendix). 10.1016/S1470-2045(11)70293-5. 22169269

[37] Bedford J.L., Hanson I.M., Hansen V.N. (2018). Comparison of forward- and back-projection in vivo EPID dosimetry for VMAT treatment of the prostate. Phys Med Biol.

[38] Williams R.J., Hinton G.E., Rumelhart D.E. (1986). Learning representations by back-propagating errors. Nature.

[39] Cho K, van Merrienboer B, Gulcehre C, Bahdanau D, Bougares F, Schwenk H, et al. Learning phrase representations using RNN encoder-decoder for statistical machine translation. arXiv:1406.1078.

[40] Kohavi R. A study of cross-validation and bootstrap for accuracy estimation and model selection. In: Proceedings of the 14th International Joint Conference on Artificial Intelligence, Montreal, Quebec, Canada, August 1995, San Francisco, CA: Morgan Kaufmann; 1995. https://www.ijcai.org/Proceedings/95-2/Papers/016.pdf.

[41] Hastie T., Tibshirani R., Friedman J. (2017). https://web.stanford.edu/%7Ehastie/Papers/ESLII.pdf.

[42] Zhuang Y., Han J., Chen L., Liu X. (2019). Dose-volume histogram prediction in volumetric modulated arc therapy for nasopharyngeal carcinomas based on uniform-intensity radiation with equal angle intervals. Phys Med Biol.

[43] Bojechko C., Ford E.C. (2015). Quantifying the performance of in vivo portal dosimetry in detecting four types of treatment parameter variations. Med Phys.

[44] Mijnheer B., Jomehzadeh A., González P., Olaciregui-Ruiz I., Rozendaal R., Shokrani P. (2018). Error detection during VMAT delivery using EPID-based 3D transit dosimetry. Phys Med.

[45] Passarge M., Fix M.K., Manser P., Stampanoni M.F.M., Siebers J.V. (2017). A Swiss cheese error detection method for real-time EPID-based quality assurance and error prevention. Med Phys.

[46] Hanson I.M., Hansen V.N., Olaciregui-Ruiz I., van Herk M. (2014). Clinical implementation and rapid commissioning of an EPID based in-vivo dosimetry system. Phys Med Biol.

[47] Mijnheer B.J., González P., Olaciregui-Ruiz I., Rozendaal R.A., van Herk M., Mans A. (2015). Overview of 3-year experience with large-scale electronic portal imaging device–based 3-dimensional transit dosimetry. Pract Radiat Oncol.

[48] Nailon W.H., Welsh D., McDonald K., Burns D., Forsyth J., Cooke G. (2019). EPID-based in vivo dosimetry using Dosimetry Check™: overview and clinical experience in a 5-yr study including breast, lung, prostate, and head and neck cancer patients. J Appl Clin Med Phys.

[49] Olaciregui-Ruiz I., Rozendaal R., van Kranen S., Mijnheer B., Mans A. (2020). The effect of the choice of patient model on the performance of in vivo 3D EPID dosimetry to detect variations in patient position and anatomy. Med Phys.

[50] Pascanu R, Mikolov T, Bengio Y. On the difficulty of training recurrent neural networks. *arXiv* 2012;*1211.5063* [*cs.LG*].

[51] Castiglioni I., Rundo L., Codari M., Di Leo G., Salvatore C., Interlenghi M. (2021). AI applications to medical images: from machine learning to deep learning. Phys Med.

[52] Huang Y., Pi Y., Ma K., Miao X., Fu S., Chen H. (2021). Virtual patient-specific quality assurance of IMRT using UNet++: classification, gamma passing rates prediction, and dose difference prediction. Front Oncol.

